# Effects of Long-Term Storage on the Chemical Composition, Antioxidant Activity, Free Amino Acid and Phenolic Profile of Traditional Vino Cotto

**DOI:** 10.3390/molecules31183268

**Published:** 2026-09-15

**Authors:** Paweł Hanus, Anna Bawoł-Król, Natalia Szarek, Przymysław Horeczy, Grażyna Jaworska

**Affiliations:** 1 Department of Food Technology and Human Nutrition, University of Rzeszów, St. Zelwerowicza 4, 35-601 Rzeszów, Poland; abawol@ur.edu.pl (A.B.-K.); przemyslawhoreczy@gmail.com (P.H.); gjaworska@ur.edu.pl (G.J.); 2 Doctoral School of the University of Rzeszów, University of Rzeszów, Rejtana 16C Street, 35-959 Rzeszów, Poland; nataliasz@dokt.ur.edu.pl

**Keywords:** vino cotto, cooked wine, sugar profile, anthocyanins, browning index, Maillard reaction, CIE L*a*b*

## Abstract

Although vino cotto is a traditional Italian cooked wine, its chemical evolution during long-term maturation remains poorly characterized. This study evaluated the physicochemical composition, phenolic profile, free amino acids, antioxidant activity, and color parameters of vino cotto from Abruzzo aged for 3, 15, and 40 years, using Trebbiano d’Abruzzo as a reference wine. Samples differing in the age of the vino cotto were characterized by marked differences in alcohol content, organic acids, sugars, total polyphenols, anthocyanins, and antioxidant activity. The total polyphenol content ranged from 0.96 to 1.56 g GAE/L, and the ABTS^•+^ activity ranged from 0.96 to 13.07 mmol Trolox/L, with the highest values observed in the 40-year-old sample. The total content of free amino acids did not differ significantly among the samples studied, although significant differences were observed among individual amino acids. Compared with Trebbiano d’Abruzzo, vino cotto showed higher levels of most phenolic compounds and greater antioxidant activity. These results indicate that the prolonged aging process, combined with the production process and other factors specific to a given batch, may result in significant differences in the chemical composition, color, and antioxidant properties of vino cotto.

## 1. Introduction

Vino cotto, also known as ‘cooked wine’, is a traditional dessert wine produced by fermenting grape must that has been previously cooked, with or without the addition of fresh must. This winemaking method is typical of certain regions in Italy, particularly Marche and Abruzzo. Although vino cotto has a long history in Italy, information on its composition remains limited [1]. Until 2000, with the exception of Marsala, it was impossible to commercially market fermented cooked grape must under Italian law. Despite these legal restrictions, vino cotto retained its traditional importance in the regions of production where it was made for home consumption. Vino cotto was recognized as a ‘Traditional Regional Product’ for the Marche and Abruzzo regions in 2000 and 2003, respectively, and has been allowed to be traded ever since [2]. Vino cotto is traditionally made from white grapes, mainly the *Trebbiano*, *Passerina*, *Montonico*, and *Moscato* varieties, and sometimes from red grapes such as *Montepulciano* and *Sangiovese* [3,4].

Vino cotto is obtained from cooked grape must and is traditionally subjected to alcoholic fermentation followed by prolonged maturation in wooden vessels, which together promote complex chemical transformations involving sugars, organic acids, phenolics, amino acids, and color-forming compounds [5,6,7,8].

Vino cotto can also be obtained by adding 10–15% sapa to the fermenting must; sapa is grape must concentrated to 30% of its original volume. Vino cotto requires a minimum of three years of aging and is characterized by its sweet taste [8].

Previous studies on vino cotto have mainly focused on selected aspects of its microbial ecology, aromatic profile, general composition, antioxidant activity, and changes associated with must cooking [1,5,6,7,8]. These studies demonstrated that vino cotto is a chemically complex product shaped by thermal processing, fermentation, and maturation. However, integrated information on long-aged vino cotto remains limited, particularly with respect to the combined evaluation of physicochemical parameters, sugar profile, free amino acids, phenolic acids, flavonoids, anthocyanins, antioxidant activity, and instrumental color parameters in samples differing markedly in maturation time.

The aim of the study was to characterize traditional Italian dessert wine vino cotto produced in the Abruzzo region, with particular emphasis on compositional differences observed among vino cotto samples aged for 3, 15, and 40 years in terms of physicochemical composition, phenolic profile, free amino acid content, antioxidant activity, and color parameters. In addition, vino cotto samples were compared with a reference white wine, Trebbiano d’Abruzzo, produced from the *Trebbiano* grape variety.

## 2. Results and Discussion

Table 1 presents the basic physicochemical parameters, sugar profile, and sulfur dioxide content of vino cotto aged for 3, 15, and 40 years, together with Trebbiano d’Abruzzo used as a reference wine. Significant differences were observed among vino cotto samples aged for 3, 15, and 40 years for most analyzed parameters, whereas free SO_2_ did not differ significantly among samples. The density of vino cotto ranged from 1.04 to 1.06 g/cm^3^ and was higher than that of Trebbiano d’Abruzzo, reflecting the high residual sugar and extract content resulting from must concentration before fermentation. Alcohol content increased from 13.35% vol in VC 3 to 15.88% vol in VC 40. This increase may be associated with progressive water loss during barrel aging, which can concentrate ethanol and other soluble constituents [9]. In contrast, Trebbiano d’Abruzzo showed a lower alcohol content, consistent with its different production technology and the absence of must cooking and prolonged concentration. The alcohol content of vino cotto was 1.43–3.96% higher by volume than that of Trebbiano d’Abruzzo white wine. A comparison of the results obtained with the data of Di Mattia et al. [5], in which the alcohol content of white wines ranged from 8–16% by volume, indicates that the values obtained in the study in question are in the upper range of the specified range. These differences are due to different production technology and specific vino cotto maturation conditions, which can affect changes in chemical composition, including an increase in alcohol content in older samples. Baiano et al. [10], on the other hand, observed a significant reduction in alcohol levels in Minutolo white wine from Puglia during the maturation process.

High acidity and low-pH environments play a key role in shaping the complex flavor profile of wine, allowing the aromatic components of the grapes to be transferred to the must as early as the pressing stage [11]. Low pH (<3.5) contributes to enhancing wine stability by inhibiting the growth of harmful bacteria and yeasts, and delays or prevents oxidation processes. It also keeps phenolic compounds in a non-ionized state, making them less susceptible to oxidation and microbial action. However, pH values below 2.9 have a negative effect on microorganisms, including *Saccharomyces cerevisiae*, leading to inhibition of their growth and delay of alcoholic fermentation [12]. Long-term maturation was also associated with changes in acidity. In the 3-year-old vino cotto studied, the pH was found to be 3.24, while in the 15- and 40-year-old wines, the values were significantly lower at 3.09 and 3.14, respectively, and were not statistically different. A different trend was observed by Xia et al. [13], who showed that there was a systematic decrease in pH values with increasing storage time of white wine in the bottle. These authors pointed out that a gradual decrease in pH is a characteristic phenomenon during the long-term maturation of wine, which may result, among other things, from the precipitation of organic acid salts and changes in the ionic balance during storage.

The trends of changes in total acidity and volatile acidity of the wines tested were the opposite of pH. After three years of maturation, the total acidity and volatile acidity of the wine tested were 8.24 g/L and 0.97 g/L, respectively. After maturation, a statistically significant (*p* < 0.001) increase in the total and volatile acidity of this wine to 9.75 and 1.27 g/L was noted. The results obtained were lower or at the lower end of the range reported by Di Mattia et al. [5] for vino cotto samples from the Abruzzo region, whose total acidity was between 9 and 19 g/L. It should be noted that wine acidity is influenced by the characteristics of the raw material, in addition to the fermentation process, related to atmospheric conditions during grape ripening [1]. One of the main reasons for excessive acidity in wine is the presence of L-malic acid, which occurs in grape in amounts ranging from 1 to 16 g/L, depending on climate, region, season and variety [12]. Organic acids are also formed through hydrolysis, biochemical metabolism, and microbial action during fermentation and aging [14]. Malic acid was found in each sample of the wine analyzed, and its content increased significantly (*p* < 0.001) as the maturation period increased, ranging from 2.85 g/L (VC 3) to 3.80 g/L (VC 40). In their study, Battistelli et al. [1] analyzed the organic acid composition of Vino cotto samples from different vintages: 1890, 1895, 1920, 1975 and 2008. Various organic acids were found, of which malic and succinic acid were predominant. The malic acid content ranged from 8.49 g/L in the 1975 vintage wine to 11.76 g/L in the 2008 sample, while the succinic acid content ranged from 4.15 g/L (1895 vintage) to 7.73 g/L (2008 vintage). The presence of citric acid at low concentrations of around 0.3 g/L, as well as tartaric and acetic acids at around 2 g/L, was also noted in all samples analyzed. Lactic acid was present only in trace amounts. The results obtained in the paper, as well as those reported by Battistelli et al. [1], indicate that the characteristic acid profile of vino cotto largely depends on the quality of the raw material, and the results of Battistelli et al. [1] suggest that it is independent of the length of its storage period.

The Trebbiano d’Abruzzo white wine had a similar pH to the 3-year-old vino cotto, but had 30% lower total acidity than it, almost three times lower volatile acidity and 2.7 times lower malic acid.

In the determination of the sugar content, fructose, glucose, and sucrose were taken into account, and the total amount of sugars was calculated on this basis. The results revealed differences in the sugar profile of the wine depending on its maturation time. The fructose content systematically increased with storage time—in 3-year wine, it was 9.45 g/L; in 15-year wine, it was 11.62 g/L; and in 40-year wine, it reached 14.74 g/L, and all the observed changes were statistically significant (*p* < 0.001). A similar trend for glucose was observed, whose concentration in 3-year-old wine was 81.19 g/L and in 40-year-old wine was 114.36 g/L. On the other hand, sucrose content was highest in 15-year-old wine (64.50 g/L) and significantly lower in 3-year-old wine (43.25 g/L) and 40-year-old wine (53.45 g/L). The total sugar content depended on the age of the wine and was 133.90 g/L in 3-year-old wine, 180.95 g/L in 15-year-old wine, and 182.55 g/L in 40-year-old wine.

A comparison of these results with the data presented in Di Mattia et al. [5], who showed that the content of reducing sugars in vino cotto is in the range of 83–350 g/L, indicates that the content of reducing sugars in the analyzed wines is at the lower end of this range. The results also suggest that the wine aging process leads to a modification of the sugar profile, in particular through a gradual increase in fructose and glucose, most likely resulting from the partial decomposition of sucrose, with the total sugar content stabilizing after about 15 years of maturation. The study showed that vino cotto has a completely different sugar profile than Trebbiano d’Abruzzo white wine. This wine showed only 1.73 g/L fructose, 3.40 g/L glucose and 3.69 g/L sucrose.

SO_2_ is a frequently used additive for the microbiological control of wine [15]. Sulfur dioxide is used as an additive to must before alcoholic fermentation begins. It inhibits the growth of undesirable yeasts of the genus *Saccharomyces*, acetic acid bacteria (AAB), and lactic acid bacteria LAB [16]. In addition to these benefits, the use of SO_2_ in winemaking can also lead to adverse effects. High concentrations of residues of this compound can affect the final taste of wine in an undesirable way [17]. For this reason, it is a requirement to include information on the label about the presence of SO_2_ when it exceeds 10 mg/L Directive 2003/89/EC [18]. According to European Union legislation, the total sulphur dioxide content of wines, other than sparkling wines and liqueur wines, may not exceed 150 mg/L for red wines and 200 mg/L for white and rosé wines [19]. However, higher limits may apply to wines with a sugar content, expressed as the sum of glucose and fructose, of not less than 5 g/L.

The contents of bound and total SO_2_ differed significantly among vino cotto samples (*p* < 0.001), whereas free SO_2_ did not (*p* = 0.068). The amount of free SO_2_ ranged from 5.41 to 5.82 mg/L. Significantly, the highest bound and total SO_2_ was found in wine aged 15 years (97.99 and 103.53 mg/L), significantly lower in wine aged for 3 years (84.01 and 89.83 mg/L), while the lowest content was found in 40-year-old wine (78.98 and 84.41 mg/L). Baiano et al. [10] observed that the total SO_2_ in fresh white wines of the Minutolo variety averaged 90.5 mg/L, while in those aged for 12 months it was higher and ranged from 129.9 to 190.1 mg/L, so also this study confirms that wine aging can lead to an increase in SO_2_ content. The Trebbiano d’Abruzzo white wine had a fairly similar amount of bound SO_2_ to the vino cotto, but had as much as nearly 7 times more free SO_2_, resulting in more than 50% higher total SO_2_ content in this wine compared to vino cotto.

Overall, the results show that the physicochemical composition of vino cotto is strongly shaped by both must concentration and long-term maturation. The combined increase in alcohol, acidity, residual sugars, and bound sulfur dioxide indicates that vino cotto evolves as a highly concentrated and chemically complex wine matrix, clearly distinct from conventional white wine produced from the same grape variety.

Free amino acids are naturally occurring nitrogenous compounds in wine and play an important role in fermentation, wine aroma development, taste formation, and chemical stability. They serve as nutrients for yeasts during alcoholic fermentation and act as precursors of higher alcohols, esters, aldehydes, and other volatile compounds that contribute to the wine bouquet [20]. In the present study, 13 free amino acids were detected in all vino cotto samples, with proline, glutamic acid, lysine, and arginine being the dominant compounds regardless of wine age (Table 2). Their presence is relevant not only from a compositional point of view but also because amino acids may contribute to the sensory profile and long-term chemical evolution of vino cotto [21].

The amino acid profile of vino cotto may be particularly important for its taste complexity. Amino acids such as proline, glycine, serine, and threonine are associated with sweet taste attributes and may enhance the perception of sweetness, which is an important characteristic of dessert wines. In contrast, leucine, valine, and phenylalanine may contribute bitter notes, whereas glutamic and aspartic acids may be associated with acidic and umami-like taste impressions [22]. Therefore, even though amino acids occur at relatively low concentrations compared with sugars and organic acids, their contribution to the overall sensory complexity of vino cotto should not be neglected, especially in the context of prolonged maturation.

The maturation period significantly affected the concentrations of several individual free amino acids. Significant differences among vino cotto samples were observed for cysteine, alanine, serine, proline, aspartic acid, glutamic acid, leucine, lysine, and arginine, with *p*-values ranging from <0.001 to 0.047. In contrast, glycine, phenylalanine, valine, and threonine did not differ significantly among the vino cotto samples. The highest concentrations of cysteine, serine, glutamic acid, and leucine were generally found in the 3-year-old vino cotto, whereas proline reached its highest level in the 15-year-old sample. Aspartic acid, lysine, and arginine showed the highest concentrations in the 40-year-old sample, suggesting that long-term maturation modifies the balance between individual amino acids rather than the total free amino acid pool.

Proline was the predominant amino acid in all vino cotto samples, ranging from 35.29 mg/L in VC 3 to 37.34 mg/L in VC 15. Glutamic acid was the second most abundant amino acid, although its concentration decreased significantly with maturation, from 19.58 mg/L in VC 3 to 18.39 mg/L in VC 40. Lysine ranged from 10.42 to 11.33 mg/L and was significantly higher in the older samples, whereas arginine increased progressively from 7.05 mg/L in VC 3 to 8.97 mg/L in VC 40. The concentrations of valine and leucine were relatively low, ranging from 0.66 to 0.89 mg/L and from 0.59 to 0.81 mg/L, respectively. The remaining amino acids, including cysteine, alanine, glycine, serine, phenylalanine, and threonine, occurred at intermediate levels, mostly between 1.37 and 3.89 mg/L.

The total free amino acid content did not differ significantly among vino cotto samples and ranged from 92.18 to 93.54 mg/L. This indicates that, despite statistically significant changes in individual amino acids, the overall free amino acid pool remained relatively stable during maturation. Such a pattern suggests that long-term aging of vino cotto does not simply lead to a general increase or decrease in free amino acids, but rather to a redistribution of individual compounds. This may result from several simultaneous processes, including peptide hydrolysis, amino acid degradation, participation in Maillard-type reactions, Strecker degradation, and interactions with carbonyl compounds formed during must cooking and wine aging.

The role of amino acids in Maillard reactions is particularly relevant for vino cotto because must heating is a key technological step in its production. During cooking and subsequent maturation, reducing sugars and reactive amino acids may participate in non-enzymatic browning reactions, leading to the formation of aroma-active and color-forming compounds [23]. Amino acids such as alanine, serine, cysteine, and lysine may contribute to the formation of aldehydes, ketones, heterocyclic compounds, and other Maillard-derived products associated with caramel, toasted, nutty, and cooked notes [23,24]. This mechanism may partly explain the characteristic sensory profile and progressive color development of long-aged vino cotto.

Compared with vino cotto, Trebbiano d’Abruzzo showed a markedly higher total free amino acid content, reaching 140.77 mg/L. This difference was mainly associated with the much higher proline concentration in the reference wine, which was approximately twice that found in vino cotto. Trebbiano d’Abruzzo also contained higher levels of arginine, lysine, phenylalanine, valine, and leucine than the vino cotto samples. These differences may be related to the absence of must cooking in conventional white wine production, differences in fermentation conditions, lower sugar concentration, and shorter or less oxidative maturation. In vino cotto, the heating of grape must and long-term aging may promote amino acid transformation and incorporation into Maillard reaction products, which could reduce the availability of some free amino acids.

The dominance of proline in both vino cotto and Trebbiano d’Abruzzo is consistent with the typical amino acid composition of grape must and wine. According to Waterhouse et al. [25], proline is one of the most abundant amino acids in grape musts, followed by other nitrogenous compounds such as arginine, valine, and alanine. The results obtained in the present study partially confirm this pattern, although the concentrations detected in vino cotto were much lower than values reported for many conventional grape musts. This may reflect the specific processing conditions of vino cotto, particularly must concentration, thermal treatment, slow fermentation, and prolonged maturation.

Overall, the results indicate that vino cotto samples differing in maturation time showed significant differences in the profile of individual free amino acids, while the total free amino acid content remained relatively stable. The observed changes suggest that amino acids participate in ongoing chemical and biochemical transformations during aging, including reactions linked to aroma formation, non-enzymatic browning, and the development of the characteristic composition of this traditional cooked wine.

The vino cotto samples differed significantly in total polyphenol content and antioxidant activity, as determined by ABTS^•+^, DPPH^•^, and FRAP assays (Table 3). Total polyphenol content increased progressively with maturation time, from 0.96 g GAE/L in VC 3 to 1.56 g GAE/L in VC 40. Trebbiano d’Abruzzo contained a lower amount of total polyphenols, 0.61 g GAE/L, which confirms the distinct chemical character of vino cotto compared with conventional white wine produced from the same grape variety.

The increase in total polyphenol content during maturation may reflect both the concentration effect associated with long-term barrel aging and the progressive release or transformation of phenolic compounds. Similar observations have been reported for selected white wines during aging. Ferreira-Lima et al. [26] observed an increase in total polyphenol content in Goethe white wines after 10 months of storage, although they also noted an initial decrease during the first two months, indicating that polyphenol evolution during storage is dynamic and depends on the balance between degradation, oxidation, polymerization, and extraction processes. Ortega et al. [27] also reported changes in total polyphenol content during the aging of white sherry wines from southern Spain over a 14-year period, including both storage under variable temperature conditions and prolonged maturation under stable conditions.

The maturation of wine is associated with numerous chemical and biochemical transformations that can affect not only the quantity but also the structure and reactivity of phenolic compounds. During prolonged aging, oxidation, hydrolysis, condensation, and polymerization reactions may occur, leading to the formation of new phenolic derivatives and polymeric pigments. These reactions can modify the response measured by the Folin–Ciocalteu assay, which reflects the reducing capacity of the sample and may therefore be influenced not only by simple phenolic compounds but also by other reducing substances formed during must cooking and wine aging. This aspect is particularly important in vino cotto, where thermal concentration of grape must may favor non-enzymatic browning, including Maillard-type reactions and melanoidin formation, which can also contribute to reducing power and antioxidant response [5,6,23,28].

The content and composition of phenolic compounds in wine are also strongly affected by grape variety, grape maturity, climatic conditions, sunlight exposure, and vinification technology [29]. Therefore, the higher total polyphenol content observed in vino cotto compared with Trebbiano d’Abruzzo should be interpreted as the combined effect of raw material composition, must concentration, thermal processing, fermentation, and long-term maturation. The cooking of grape must may concentrate phenolic constituents and promote their transformation, while barrel aging may further modify their profile through oxidation and condensation reactions.

The antioxidant capacity of the wines was evaluated using three complementary assays: ABTS^•+^, DPPH^•^, and FRAP. The ABTS^•+^ and DPPH^•^ methods are based on the ability of antioxidants to neutralize stable free radicals, whereas the FRAP assay measures the reducing power of the sample through the reduction of ferric ions [30,31]. The use of these three methods provides a broader assessment of antioxidant potential because different antioxidant compounds may respond differently depending on the reaction mechanism and assay conditions.

A particularly strong increase was observed for ABTS^•+^ radical scavenging activity, which rose from 0.96 mmol Trolox/L in VC 3 to 13.07 mmol Trolox/L in VC 40. Thus, the ABTS^•+^ value of the 40-year-old vino cotto was approximately 13 times higher than that of the 3-year-old sample. The much stronger increase in ABTS^•+^ activity compared with DPPH^•^, FRAP, and total polyphenols indicates that the ABTS^•+^ assay may have responded to a broader range of matrix constituents. In addition to phenolic compounds, cooked and long-aged wine matrices may contain Maillard reaction products, melanoidins, sulfites, polymeric pigments, and other hydrophilic reducing compounds that can contribute to the ABTS^•+^ radical cation decolorization response. Therefore, the high ABTS^•+^ value observed in VC 40 should be interpreted as an assay-specific antioxidant response of a complex aged matrix, rather than as evidence of a proportional increase in simple phenolic compounds alone.

A similar, although less pronounced, trend was observed for DPPH^•^ and FRAP. The DPPH^•^ radical scavenging activity increased significantly from 2.14 mmol Trolox/L in VC 3 to 3.68 mmol Trolox/L in VC 40, whereas FRAP values increased from 3.35 to 5.72 mmol Trolox/L over the same maturation period. These results indicate that long-term maturation enhanced both radical scavenging capacity and reducing power. The highest antioxidant activity was consistently observed in VC 40, while VC 3 showed the lowest values in all assays. These results suggest that the higher antioxidant potential observed in the older vino cotto samples may be associated with prolonged maturation, although vintage, raw material composition, production conditions, and batch-specific factors may also have contributed to the observed differences.

The observed increase in antioxidant activity is consistent with the progressive enrichment of vino cotto in phenolic compounds; however, the magnitude of the ABTS^•+^ response indicates that additional compounds formed during heating and aging may also contribute. In contrast, Baiano et al. [10] reported a decrease in antioxidant activity during the maturation of Minutolo white wine from the Puglia region, which highlights that the evolution of antioxidant capacity depends strongly on wine type, production technology, aging conditions, and initial phenolic composition. Unlike conventional white wine, vino cotto is produced from cooked grape must and undergoes long-term maturation, which may promote the formation and accumulation of antioxidant compounds not typically present at comparable levels in standard white wines.

Trebbiano d’Abruzzo showed lower antioxidant activity than vino cotto in the DPPH^•^ and FRAP assays and a similar ABTS^•+^ value to the 3-year-old vino cotto. These results suggest that the older vino cotto samples had higher antioxidant potential, which may be associated with prolonged maturation, although production conditions, raw material composition, vintage, and batch-specific factors may also have contributed to the observed differences. The lower antioxidant potential of Trebbiano d’Abruzzo may be related to the absence of must cooking, lower total polyphenol content, lower sugar concentration, and shorter or less oxidative aging compared with vino cotto.

The total polyphenol content and antioxidant activity of vino cotto were strongly associated with its phenolic profile. In particular, high correlation coefficients were observed for selected phenolic acids, including gallic acid, ethyl gallate, and protocatechuic acid, as well as for flavonoids such as procyanidin B2, quercetin, quercetin 3-O-glucoside, kaempferol 3-O-glucoside, myricetin, and most anthocyanins. The correlation coefficients ranged from 0.74 to 0.99, suggesting that these compounds may contribute substantially to the antioxidant properties of vino cotto. However, these relationships should be interpreted with caution because antioxidant activity is the result of combined and potentially synergistic interactions among several groups of compounds, including phenolics, Maillard reaction products, and other reducing constituents formed during processing and aging.

Overall, the results indicate that vino cotto samples differing in age were characterized by marked differences in antioxidant potential and total polyphenol content, which may be associated with prolonged maturation as well as production- and batch-specific factors. The particularly strong ABTS^•+^ response of the 40-year-old sample suggests that prolonged aging promotes the formation or accumulation of compounds with high radical scavenging capacity. These findings support the hypothesis that the distinctive antioxidant properties of vino cotto result from the combined effects of grape must concentration, thermal processing, phenolic transformations, and long-term maturation.

Table 4 summarizes the quinic acid and phenolic profile of vino cotto, which were determined using high-performance liquid chromatography and ultra-performance liquid chromatography coupled with mass spectrometry (UPLC-MS).

Among the compounds determined, quinic acid was identified as a non-phenolic organic acid, whereas gallic acid, ethyl gallate, protocatechuic acid, caftaric acid, ferulic acid, and caffeic acid were classified as phenolic acids. The concentrations of these compounds were significantly affected by the age of vino cotto, indicating that long-term maturation strongly modifies the phenolic acid profile of this traditional cooked wine.

Quinic acid was the dominant non-phenolic organic acid in all vino cotto samples and increased significantly from 182.00 mg/L in VC 3 to 304.88 mg/L in VC 40. This increase may be related to the hydrolysis of phenolic esters and other conjugated compounds during aging. Under acidic wine conditions and in the presence of oxygen, ester hydrolysis and oxidative transformations may promote the release and accumulation of simpler phenolic and organic acids. The high stability of quinic acid in the wine matrix may also contribute to its progressive accumulation during long-term maturation [32]. The presence of quinic acid in wine has also been reported by Wen et al. [33], who found this compound, together with citric acid, among the main organic acids in wines produced from *Actinidia arguta*.

A similar increasing trend was observed for selected hydroxybenzoic acid derivatives. Gallic acid increased from 44.45 mg/L in VC 3 to 69.85 mg/L in VC 40, while protocatechuic acid increased from 9.24 to 27.45 mg/L. Ethyl gallate also increased markedly, from 4.32 mg/L in VC 3 to 11.91 mg/L in VC 40. These changes may indicate the progressive hydrolysis of more complex phenolic structures, including tannins, galloylated compounds, and phenolic esters, leading to the formation of simpler hydroxybenzoic acid derivatives [34]. The accumulation of these compounds is particularly relevant because gallic acid, protocatechuic acid, and ethyl gallate are known to contribute to the antioxidant potential and chemical reactivity of wine.

Different behavior was observed for hydroxycinnamic acid derivatives. Caftaric acid decreased significantly from 3.71 mg/L in VC 3 to 2.95 mg/L in VC 40, which may be associated with its oxidation, hydrolysis, or participation in condensation reactions during aging. Caffeic acid showed a non-linear pattern, with the lowest concentration in VC 15 and the highest in VC 40. This may reflect secondary release from esterified forms, transformation of caftaric acid, or further involvement in oxidative reactions. Ferulic acid was detected only in very small amounts, with the highest value in VC 15. Such behavior is consistent with the known susceptibility of hydroxycinnamic acids to oxidation, ester hydrolysis, and reactions with glutathione, sulfur compounds, and other wine matrix constituents [35,36].

The total content of phenolic acids increased significantly with the age of vino cotto, from 66.16 mg/L in VC 3 to 117.07 mg/L in VC 40. This confirms that long-term maturation promotes the accumulation or release of selected phenolic acids. However, this increase should not be interpreted only as a simple concentration effect caused by water loss during barrel aging. It is more likely the result of several simultaneous processes, including hydrolysis of bound phenolics, oxidation of phenolic precursors, transformation of hydroxycinnamic acids, and formation of new derivatives during prolonged storage.

The phenolic profile of Trebbiano d’Abruzzo differed markedly from that of vino cotto. In the reference wine, gallic acid was the predominant phenolic acid (64.26 mg/L), while quinic acid, classified separately as a non-phenolic organic acid, reached 36.92 mg/L. Caftaric acid was also present at a relatively high concentration, 10.27 mg/L, whereas protocatechuic acid and caffeic acid occurred at much lower levels [37]. The total phenolic acid content of Trebbiano d’Abruzzo was 81.61 mg/L. This value was higher than that observed in VC 3 (66.16 mg/L), but lower than in VC 15 (95.86 mg/L) and VC 40 (117.07 mg/L). The largest differences in favor of vino cotto were observed for protocatechuic acid and quinic acid, whose concentrations were many times higher than in the reference wine. In contrast, Trebbiano d’Abruzzo contained more caftaric acid than vino cotto, which may reflect differences in vinification technology, lower thermal transformation, and limited long-term oxidative maturation [38,39].

The flavonoid fraction included flavanols, represented mainly by procyanidins and their derivatives, flavonols represented by quercetin, kaempferol derivatives, and myricetin, as well as hesperetin, which belongs to flavanones. Anthocyanins are discussed separately in Table 5. The flavonoid profile showed more diverse changes during maturation than the phenolic acid fraction, suggesting that flavonoids are more susceptible to oxidation, polymerization, and degradation during long-term aging.

Among flavanols, procyanidin B1 was the dominant compound in VC 3 and VC 15, but its concentration decreased markedly in VC 40. This decrease may indicate degradation, oxidation, or incorporation into polymeric structures during prolonged maturation. In contrast, procyanidin B2 increased from 0.04 mg/L in VC 3 to 0.69 mg/L in VC 40, suggesting that individual procyanidins differ in their stability and transformation pathways during aging. Procyanidin dimers were detected only in VC 3 and were not detected in older vino cotto samples, which further supports the hypothesis that flavanols undergo progressive structural transformation during maturation. Jouin et al. [40] reported that the oxidation behavior of procyanidins depends strongly on their structure, with some oligomeric forms showing rapid degradation and others remaining more stable even after long-term storage.

The total flavanol content was highest in VC 15 and VC 3, while VC 40 showed a much lower value. This indicates that, unlike phenolic acids, flavanols do not accumulate during prolonged maturation. Instead, they may be transformed into more complex polymeric compounds that are not quantified as individual procyanidins under the analytical conditions applied. Such transformations may contribute to changes in color, astringency, bitterness, and antioxidant properties of vino cotto.

Trebbiano d’Abruzzo contained 7.97 mg/L of total flavanols, with procyanidin B1 being the predominant compound. Compared with vino cotto, the reference wine had lower total flavanol content than VC 3 and VC 15, but higher than VC 40. This pattern suggests that the younger and intermediate-aged vino cotto samples retained more monomeric and oligomeric flavanol structures, whereas the 40-year-old sample underwent more advanced flavanol transformation. These differences may be associated with the specific conditions of vino cotto production, including must heating, high sugar concentration, oxygen exposure, and prolonged barrel aging.

Flavonols were present at low concentrations in all vino cotto samples. The total flavonol content ranged from 0.26 to 0.55 mg/L and did not show a simple linear trend with aging. Quercetin was not detected in VC 3 but was present in VC 15 and VC 40, increasing to 0.13 mg/L in the oldest sample. This may suggest the gradual hydrolysis of quercetin glycosides or release of quercetin from more complex forms during maturation. Lanati et al. [41] indicated that quercetin 3-O-glucoside can be hydrolyzed during wine maturation, leading to an increase in free quercetin. In the present study, quercetin 3-O-glucoside remained at very low levels, whereas free quercetin increased with age, which supports this interpretation.

Myricetin also increased from 0.01 mg/L in VC 3 to 0.04 mg/L in VC 15 and VC 40, while kaempferol 3-O-glucoside remained relatively stable, ranging from 0.16 to 0.18 mg/L. This may indicate higher stability of kaempferol glycosides under the conditions of vino cotto maturation. Luteolin was detected only in VC 3 and was not found in the older samples, which may reflect its degradation or transformation during aging. Overall, the low concentration of flavonols in vino cotto may be related to the use of white grape varieties, limited extraction from kins, thermal processing of must, and long-term oxidative conditions.

In Trebbiano d’Abruzzo, flavonols were also present in trace amounts. Kaempferol 3-O-glucoside was detected at 0.15 mg/L, while quercetin, quercetin 3-O-glucoside, and myricetin were absent or present at very low levels. The total flavonol content of Trebbiano d’Abruzzo was 0.15 mg/L, which was lower than in vino cotto. This confirms that, despite the generally low flavonol levels in white wines, the production and maturation conditions of vino cotto may favor the retention or formation of certain flavonol derivatives [39,41,42].

Hesperetin was the only flavanone identified in vino cotto. Its concentration decreased strongly with maturation time, from 2.07 mg/L in VC 3 to 0.20 mg/L in VC 15, and it was not detected in VC 40. This indicates that hesperetin is relatively unstable during prolonged maturation and may undergo degradation, oxidation, or transformation into compounds not detected under the applied analytical conditions. In Trebbiano d’Abruzzo, hesperetin was present at 0.94 mg/L, which was lower than in VC 3 but higher than in VC 15 and VC 40. This comparison suggests that the fate of hesperetin is strongly affected by both processing and aging conditions [42].

The comparison with Trebbiano d’Abruzzo confirms that vino cotto has a phenolic profile clearly distinct from conventional white wine produced from the same grape variety. The reference wine was characterized mainly by simple phenolic acids, especially gallic and caftaric acids, together with a relatively low content of flavonols and flavanols. In contrast, vino cotto showed much higher levels of total phenolic acids, particularly quinic, protocatechuic, and ethyl gallate, as well as marked age-related changes in flavanol and flavonol composition. These differences are likely associated with the cooking of grape must, concentration of soluble compounds, thermal and oxidative transformations, and long-term barrel maturation [39,43].

Phenolic compounds may also influence Maillard-type reactions occurring in vino cotto. In food systems, the main substrates of the Maillard reaction are reducing sugars and amino compounds, while polyphenols can modulate the reaction by interacting with amino acids, sugars, α-dicarbonyl compounds, and Amadori rearrangement products [44]. These interactions may lead to the formation of phenolic–Maillard adducts and may either inhibit or redirect the formation of melanoidins, depending on the structure of the phenolic compounds and the processing conditions [45]. Hydroxycinnamic acids, flavan-3-ols, flavonoids, and tannins differ in their reactivity, which is influenced by the number and position of hydroxyl and methoxyl groups and by the structure of their side chains. For example, ferulic acid may show stronger inhibitory effects than caffeic acid because of its greater stability and ability to form complexes with Maillard intermediates [46].

In the context of vino cotto, the simultaneous presence of sugars, amino acids, phenolic compounds, acidic pH, and oxygen creates favorable conditions for complex non-enzymatic browning and oxidation reactions during must cooking and subsequent maturation. The observed changes in quinic acid, gallic acid, ethyl gallate, protocatechuic acid, caftaric acid, and total phenolic acids may therefore be linked not only to phenolic hydrolysis and oxidation, but also to interactions with Maillard reaction intermediates and amino acids. Strong correlations were found between selected phenolic compounds and reactive amino acids, especially lysine, arginine, and cysteine, with correlation coefficients ranging from −0.78 to −0.99 and from 0.81 to 0.99. These relationships suggest that phenolic compounds and amino acids may jointly participate in the chemical evolution of vino cotto during prolonged aging.

Overall, the results demonstrate that long-term maturation strongly modifies the phenolic profile of vino cotto. Quinic acid, reported separately as a non-phenolic organic acid, increased with aging, whereas among phenolic acids, protocatechuic acid, gallic acid, and ethyl gallate were the most relevant compounds contributing to the observed changes. These changes indicate that vino cotto develops as a chemically complex matrix in which must cooking, oxidative aging, phenolic transformations, and Maillard-type reactions jointly shape the final composition, antioxidant potential, color development, and sensory character of the wine.

The anthocyanin profile of vino cotto is presented in Table 5. Five main anthocyanidin aglycone groups were identified: delphinidin, cyanidin, petunidin, peonidin, and malvidin. These compounds occurred mainly as simple glucosides, diglucosides, and acylated glucosides. Although anthocyanins are usually associated with red wines, their presence in vino cotto may result from the specific raw material and processing conditions used in traditional production, including possible contact with grape skins, the use of grape varieties containing low amounts of pigments, must concentration, and long-term maturation. In conventional white wines, anthocyanins are generally absent or present only in trace amounts because of the limited extraction from grape skins and the use of white grape varieties [47].

Despite the known instability of anthocyanins during wine aging, the total anthocyanin content in vino cotto increased significantly with maturation time. The total anthocyanin concentration increased from 7.51 mg/L in VC 3 to 9.71 mg/L in VC 15 and 15.62 mg/L in VC 40. Thus, the 40-year-old vino cotto contained more than twice as much total anthocyanins as the 3-year-old sample. This trend suggests that long-term maturation of vino cotto may favor either the retention and concentration of selected anthocyanins or the formation of more stable anthocyanin-derived pigments. The acidic pH of vino cotto, generally within the range favorable for anthocyanin stability, may also contribute to the persistence of these compounds during aging [48].

Among delphinidin derivatives, delphinidin 3-O-glucoside-5-O-glucoside, delphinidin 3-O-glucoside, delphinidin 3-O-(6″-O-acetyl)-glucoside, and delphinidin 3-O-(6″-caffeoyl)-glucoside were identified. The total delphinidin content increased significantly from 1.38 mg/L in VC 3 to 2.89 mg/L in VC 40. The strongest increase was observed for delphinidin 3-O-glucoside, whose concentration more than doubled during maturation. This indicates that delphinidin derivatives were progressively enriched or retained in the wine matrix during long-term aging.

Cyanidin derivatives were represented by cyanidin 3-O-glucoside-5-O-glucoside, cyanidin 3-O-galactoside, cyanidin 3-O-glucoside, cyanidin 3-O-(6″-O-coumaryl)-glucoside, and cyanidin 3-O-(6″-O-acetyl)-glucoside. The total cyanidin content increased significantly from 1.22 mg/L in VC 3 to 1.62 mg/L in VC 15 and 2.33 mg/L in VC 40. In particular, cyanidin 3-O-glucoside was not detected in the youngest vino cotto sample but was present in VC 15 and reached 0.93 mg/L in VC 40. The acetylated cyanidin derivative was present in all samples and showed a slight but statistically significant increase during maturation, from 0.41 mg/L in VC 3 and VC 15 to 0.44 mg/L in VC 40. These results suggest that cyanidin-derived pigments are progressively retained, released, or transformed during long-term maturation. The presence of acetylated and coumaroylated forms may also contribute to greater pigment stability in the acidic and phenolic-rich matrix of vino cotto.

Petunidin derivatives were identified as petunidin 3-O-glucoside-5-O-glucoside, petunidin 3-O-glucoside, petunidin 3-O-(6″-O-acetyl)-glucoside-5-O-glucoside, and petunidin 3-O-(6″-O-coumaryl)-glucoside. The total petunidin content increased from 1.36 mg/L in VC 3 to 1.90 mg/L in VC 40. However, the individual compounds showed different patterns. Petunidin 3-O-glucoside-5-O-glucoside and petunidin 3-O-glucoside increased with maturation, whereas petunidin 3-O-(6″-O-coumaryl)-glucoside was detected only in VC 3. This suggests that some acylated forms may be less stable during prolonged maturation or may be transformed into other pigment structures.

Peonidin derivatives occurred in three forms: peonidin 3-O-glucoside-5-O-glucoside, peonidin 3-O-glucoside, and peonidin 3-O-(6″-O-coumaryl)-glucoside. The total peonidin content increased moderately from 1.10 mg/L in VC 3 to 1.40 mg/L in VC 40. Compared with delphinidins, cyanidins, and malvidins, the increase in peonidins was less pronounced, and not all individual peonidin derivatives differed significantly among samples. This may indicate relatively higher stability of some peonidin derivatives or a lower degree of transformation during maturation.

Malvidins represented the dominant group of anthocyanins in vino cotto. This is consistent with the general anthocyanin profile of grape-derived products, as malvidin derivatives are usually among the most abundant and stable anthocyanins in wines, particularly red wines [49,50]. In vino cotto, malvidin 3-O-glucoside-5-O-glucoside, malvidin 3-O-glucoside, and malvidin 3-O-(6″-O-acetyl)-glucoside were detected. The total malvidin content increased markedly from 2.43 mg/L in VC 3 to 7.11 mg/L in VC 40. The most pronounced change was observed for malvidin 3-O-(6″-O-acetyl)-glucoside, which increased from 0.94 mg/L in VC 3 to 5.21 mg/L in VC 40. This strong increase suggests that malvidin derivatives may play a particularly important role in the pigment profile of long-aged vino cotto.

The predominance of malvidins and the increase in total anthocyanins may contribute to the color evolution of vino cotto during maturation. However, the color of long-aged cooked wines is not determined by anthocyanins alone. It is also shaped by oxidation reactions, polymeric pigments, condensation products between anthocyanins and flavanols, and Maillard-derived brown pigments formed during must heating and aging. Therefore, the increase in anthocyanin-related compounds should be considered together with browning reactions and phenolic transformations, which jointly determine the final color characteristics of vino cotto.

Trebbiano d’Abruzzo contained only 2.23 mg/L of total anthocyanins, which was more than three times lower than in the youngest vino cotto sample and approximately seven times lower than in VC 40. This confirms the distinct pigment profile of vino cotto compared with the reference white wine. The low anthocyanin content of Trebbiano d’Abruzzo is consistent with conventional white wine production, where limited skin contact strongly restricts pigment extraction. In contrast, the higher anthocyanin content of vino cotto may reflect the traditional production process, concentration of grape must, possible extraction from grape solids, and long-term formation or retention of anthocyanin-derived pigments.

The significant increase in total anthocyanins during maturation is noteworthy because anthocyanins are generally regarded as unstable during long-term storage. In vino cotto, this behavior may be explained by the specific chemical environment of the product. Low pH, high sugar concentration, high total extract, phenolic interactions, and the presence of acetylated and coumaroylated anthocyanin forms may enhance pigment stability. Moreover, during aging, anthocyanins can react with flavanols, phenolic acids, aldehydes, and other oxidation products to form more stable polymeric pigments. Such compounds may be detected as anthocyanin-related derivatives and may contribute to the apparent increase in anthocyanin content.

Overall, the results show that vino cotto contains a diverse anthocyanin profile despite being a traditional cooked dessert wine often produced from white grape varieties. Long-term maturation significantly increased the total content of most anthocyanin groups, especially malvidins, delphinidins, and cyanidins. These changes suggest that pigment evolution in vino cotto is governed not only by degradation, but also by concentration, stabilization, and transformation reactions. The anthocyanin profile therefore contributes to the distinctive color development and chemical complexity of long-aged vino cotto.

Color analysis using the CIE L*a*b* system revealed marked non-linear changes in the color parameters of vino cotto depending on maturation time. The 3-year-old wine showed low lightness (L* = 25.74) and high positive a* and b* values (a* = 34.05, b* = 44.16), indicating an intense amber to orange-brown color with a strong contribution of both red and yellow tones. In the 15-year-old wine, L* decreased markedly to 3.69, while a* and b* also decreased to 18.56 and 6.37, respectively. After 40 years of maturation, L* increased to 13.20, while a* and b* increased to 31.24 and 22.74, respectively, indicating that the changes in lightness and color coordinates did not follow a simple progressive darkening pattern. The results presented in Table 6 indicate that vino cotto undergoes significant color transformation during maturation. These changes should be interpreted primarily as oxidative browning and pigment evolution rather than only as classical pinking, which is usually described as an undesirable phenomenon in conventional white wines produced from white grapes. Pinking is associated with the development of pink or reddish hues in white wines and is often linked to the oxidation of phenolic compounds and pigment precursors [51]. Although similar oxidative mechanisms may contribute to the color changes observed in vino cotto, the color development of this product is also strongly influenced by must cooking, concentration of soluble compounds, long-term maturation, Maillard-type reactions, and the formation of brown polymeric pigments. Therefore, in vino cotto, reddish-brown color formation should be considered part of the characteristic chemical evolution of the product rather than only a technological defect.

The results of color analysis, including hue angle, chroma, and browning index (BI), further confirmed the non-linear evolution of color during maturation. Hue angle values were 52.36° in VC 3, 18.93° in VC 15, and 36.06° in VC 40, while Trebbiano d’Abruzzo showed a much higher hue angle of 103.79°. The hue angle of VC 3 indicates a dominant orange-yellow/brown hue, whereas the lower value observed in VC 15 suggests a shift toward a darker red-brown tone. The intermediate value recorded for VC 40 indicates a partial shift back toward a more balanced orange-brown hue. In contrast, the hue angle of Trebbiano d’Abruzzo was typical of a much lighter white wine with a dominant yellow-green component. These differences clearly separate vino cotto from the reference white wine and confirm the impact of must cooking and long-term maturation on color development.

Similar changes in color parameters have been reported for sweet wines such as *Pedro Ximénez* (PX), in which hue angle and chromatic characteristics changed during aging as a result of phenolic oxidation, pigment transformation, and the formation of brown compounds [52]. In the present study, the correlation analysis between hue angle and phenolic compounds also indicated the contribution of phenolic transformations to vino cotto color. Hue angle was strongly correlated with ferulic acid (r = 0.97) and showed a strong inverse correlation with caffeic acid (r = −0.91). These relationships suggest that hydroxycinnamic acid transformations may participate in color shifts during maturation. The oxidation of phenolic compounds, including o- and p-dihydroxyphenols, can lead to the formation of reactive quinones, which may react with nucleophiles such as glutathione, amino acids, and other phenols, contributing to the formation of new pigments and polymeric oxidation products [53,54].

The chroma parameter, which reflects color saturation, was highest in VC 3 (55.76), decreased markedly in VC 15 (19.62), and reached an intermediate value in VC 40 (38.64). This indicates that the youngest vino cotto had the most saturated color, while the 15-year-old sample was the darkest and least saturated. The increase in chroma observed again in VC 40 suggests further formation or stabilization of colored compounds during prolonged aging. A similar non-linear behavior of chromaticity has been described in PX wines, where changes in color saturation during maturation were attributed to the formation and transformation of phenolic oxidation products [52].

The evolution of chroma may be associated with transformations of flavanols, hydroxycinnamic acids, and other phenolic constituents. Cheynier et al. [55] attributed wine browning partly to the oxidation of hydroxycinnamoyl tartaric acids, including trans-caffeoyl tartrate and p-coumaroyl derivatives. These authors also suggested that browning may result not only from direct oxidation reactions but also from acid-catalyzed cleavage of procyanidin C–C bonds, followed by recombination of released oligomers and carbocations into larger polymeric structures capable of interacting with macromolecules such as proteins. In the present study, chroma was strongly correlated with selected phenolic compounds, including gallic acid (r = −0.81), ferulic acid (r = −0.92), caffeic acid (r = 0.83), procyanidin dimer (r = 0.85), luteolin (r = 0.85), and hesperetin (r = 0.80). These correlations support the role of phenolic transformations in shaping the saturation and intensity of vino cotto color. 

The browning index reached very high values in vino cotto, confirming the intense browning associated with this product. The highest BI value was recorded in VC 3 (812.54), while lower values were found in VC 15 (539.14) and VC 40 (668.19). These results suggest that intensive browning occurs already during the early stages of vino cotto production, most likely as a consequence of must heating and concentration. The subsequent changes in BI during maturation indicate that browning compounds continue to undergo transformation, polymerization, precipitation, or interaction with other wine constituents during aging.

In the study by Serratosa et al. [52], browning in sweet wines was assessed using absorbance at 420 nm (A_420_), which increased during aging and reached high values in Solera wines. The authors attributed this effect to phenolic oxidation, especially involving flavonols, and to the formation of melanoidins and brown polymers through Maillard reactions and condensation reactions between phenolic compounds and aldehydes. A similar mechanism may occur in vino cotto, where high sugar concentration, amino acids, phenolic compounds, acidic pH, oxygen exposure, and thermal treatment create favorable conditions for non-enzymatic browning and pigment polymerization.

In the present study, the BI parameter was inversely correlated with selected flavonols, including quercetin, quercetin 3-O-glucoside, kaempferol 3-O-glucoside, and myricetin, although a strong correlation was observed mainly for myricetin (r = −0.80). These relationships should be interpreted with caution because browning in vino cotto is probably not controlled by a single group of compounds. Instead, it likely results from the combined effects of phenolic oxidation, anthocyanin-derived pigment formation, Maillard-type reactions, melanoidin formation, and polymerization of phenolic compounds.

Both the present results and the findings reported by Serratosa et al. [52] support the usefulness of CIE L*a*b* parameters, hue angle, chroma, and browning index as sensitive indicators of chemical changes occurring during wine maturation. In vino cotto, high BI values and marked variation in hue angle and chroma indicate advanced oxidative and non-enzymatic browning processes. It should also be emphasized that the color parameters of Trebbiano d’Abruzzo were completely different from those of vino cotto. The reference wine had very high lightness (L* = 88.77), negative a* value (−2.29), low b* value (9.31), low chroma (9.58), and low browning index (8.79), confirming its bright white-wine character. In contrast, vino cotto developed a much darker amber to reddish-brown color profile, shaped by must cooking, phenolic transformations, Maillard-type reactions, and long-term maturation.

## 3. Materials and Methods

### 3.1. Material

The research material was obtained from the town of Roccamontepiano (42.243178 N, 14.128782 E) located in the Abruzzo region, that is, central Italy. The wine samples were obtained in cooperation with the local Association of Vino Cotto Producers ’Associazione Produttori Vino Cotto d’Abruzzo’. According to traditional production practices described for vino cotto, grape must is heated in copper boilers until it becomes concentrated and darker, usually reaching a sugar concentration of approximately 40–90%. The concentration process is carried out at sub-boiling temperatures, typically 80–95 °C, and may last up to 48 h. Fresh must may then be added to the cooked must in proportions depending on local practice, followed by slow alcoholic fermentation carried out by indigenous yeasts. After fermentation, vino cotto is transferred to wooden vessels for prolonged maturation [5,6,7,8]. This production background is provided to characterize the type of traditional product analyzed in the present study. For each maturation variant, three 200 mL bottles of traditionally produced vino cotto aged for 3, 15, or 40 years were analyzed. It should be noted that the three bottles within each maturation variant originated from the same production batch and were therefore treated as analytical replicates. Consequently, the comparison among the 3-, 15-, and 40-year-old vino cotto samples should be interpreted as an age-associated comparison of the analyzed samples rather than as a fully controlled longitudinal assessment of storage time alone. Vintage effects, raw material composition, production conditions, vessel/barrel characteristics, and other batch-specific factors may also have contributed to the observed differences. In addition, a white wine produced from uncooked must made from the Trebbiano d’Abruzzo grape variety—the variety used to produce vino cotto in the Abruzzo region—was included as a reference sample for comparative purposes. The sample was used to provide a comparative matrix for evaluating the chemical specificity of vino cotto.

### 3.2. Determination of Density

The density of wine samples was determined using a calibrated 25 mL glass pycnometer at 20 °C and expressed as g/cm^3^ according to OIV-MA-AS2-01A [56]. Excess carbon dioxide was removed from the sample by stirring and filtering under reduced pressure. Each sample was analyzed in triplicate.

### 3.3. Determination of Alcohol Content

The alcohol content of wine samples was determined according to OIV-MA-AS312-01A [57] by the distillation of the alkalized wine and pycnometric determination of the density of the resulting distillate.

The distillation was carried out using a standard laboratory steam distillation apparatus. Samples were alkalized with calcium hydroxide suspension (2 M) and subjected to steam distillation. The density of the resulting distillate was determined at 20 °C. The alcohol content was calculated using alcoholometric tables and expressed as % vol. Each sample was analyzed in triplicate.

### 3.4. Determination of Total Acidity and pH

Total acidity was determined by potentiometric titration (OIV-MA-AS313-01) [58]. The pH was measured potentiometrically, and titratable acidity (TA) was determined by titration, using 0.1 N NaOH to reach an end point of pH 7.0. This process was carried out using an automated pH titration system, TitroLine 7500 KF (Schott, Mainz, Germany). The results were expressed as grams of tartaric acid per liter g/L. The analysis was performed in triplicate.

### 3.5. Determination of Volatile Acidity

Volatile acidity was determined by distillation of volatile acids from the test sample with an external steam supply from a steam generator according to the appropriate OIV method OIV-MA-AS313-02 [59]. The resulting distillate was titrated hot with a standard NaOH solution against phenolphthalein as an indicator. Carbon dioxide was removed from the fermented wine beverage, whereas the acidity formed by free and bound sulfur dioxide was subtracted from the acidity of the distillate. The result was expressed as g acetic acid/L. Each sample was analyzed in triplicate.

### 3.6. Determination of L-Malic Acid

L-malic acid (L-malate) was determined enzymatically according to OIV-MA-AS313-11 [60] using an L-Malic Acid Assay Kit, Analyser Format (K-LMALAF; Megazyme, Bray, Co., Ltd., Wicklow, Ireland). In this method, L-malate is oxidized by nicotinamide adenine dinucleotide (NAD^+^) to oxaloacetate in a reaction catalyzed by L-malate dehydrogenase (L-MDH), and the amount of NADH formed is proportional to the amount of L-malate present in the sample. Absorbance was measured at 340 nm using a UV-1900 UV–Vis spectrophotometer (Shimadzu Corporation, Kyoto, Japan). The results were expressed as g/L. Each sample was analyzed in triplicate.

### 3.7. Identification and Quantification of Glucose, Fructose and Sucrose

Glucose, fructose, and sucrose were determined by HPLC using a SYKAM high-performance liquid chromatograph (Sykam GmbH, Eresing, Germany), controlled by Clarity software version 6.1 (DataApex Ltd., Prague, Czech Republic). The separation process was performed on a Cosmosil SUGAR-D column (4.6 mm × 250 mm; Nacalai Tesque, Inc., Kyoto, Japan) and the elution was carried out at a flow rate of 1 mL/min, a maximum pressure of 100 bar and a temperature of 35 ± 5 °C. A 20 µL sample was used for analysis. The mobile phase consisted of acetonitrile (phase A: 75%) and water (phase B: 25%). Quantitative analysis was carried out using external calibration curves prepared from fructose, glucose, and sucrose standards. Each wine sample was analyzed in triplicate. Results were presented in g/L to match the units reported in the results tables.

### 3.8. Determination of the Free and Bound Sulfur Dioxide Content

In the wine samples tested, the content of free and bound SO_2_ was determined using the iodometric method (OIV-MA-AS323-04B) [61]. Free sulfur dioxide was determined by direct titration with iodine. The bound sulfur dioxide was determined after alkaline hydrolysis. The sum of free and bound sulfur dioxide was used to calculate the total sulfur dioxide content. The result was expressed as mg/L. Each sample was analyzed in triplicate.

### 3.9. Preparation of Wine Samples for Chromatographic and Spectrophotometric Analyses

The wine was centrifuged in an MPW-260R laboratory centrifuge (MPW Med. Instruments, Warsaw, Poland) at 5500 rpm (4227× *g*) for 5 min and then filtered through PTFE syringe filters with a pore size of 0.45 µm before analysis.

### 3.10. Identification and Quantification of Free Amino Acids

UHPLC analysis was performed using a Waters ACQUITY UPLC system (Waters, Milford, MA, USA), which is equipped with a binary solvent delivery system and autosampler. The UHPLC separation process was carried out on an AccQ-Tag Ultra C 18 column (4.6 mm × 150 mm, 2.5 μm, Waters), which was equipped with a 2.5 μm AccQ-Tag Ultra C 18 precolumn. The mobile phase consisted of solvent A (water, 10 mM ammonium formate and 0.15% formic acid, pH 3.0) and solvent B (acetonitrile, 2 mM ammonium formate and 0.15% formic acid), with an elution gradient: 0–6 min, 15–20% A; 6–10 min, 20–30% A; 10–12 min, 30–40% A. After this, the column was equilibrated for 6 min under initial conditions. The mobile phase flow rate was 0.4 mL/min and the column temperature was maintained at 35 °C. Between injections, the system was washed with weak (20% acetonitrile) and strong (80% acetonitrile) solvents. The injection volume was 2 μL. The eluent from the column was directed to the mass spectrometer. All analyzes were performed using MassLynx XS software (version 4.1; Waters, Milford, MA, USA). The analysis was performed in triplicate.

The quantification of free amino acids in wine samples was determined using the Amino Acids Mix Solution standard Sigma Aldrich (MERCK, Darmstadt, Germany).

### 3.11. Identification and Quantification of Polyphenolic Compounds

High-performance liquid chromatography (HPLC) and ultra-high-performance liquid chromatography (UPLC) were employed to determine the content of low-molecular-weight polyphenols in wines. Flavonoids, anthocyanins, and phenolic acids were identified using HPLC-DAD (High-Performance Liquid Chromatography with Diode-Array Detection) and UPLC-ESI-MS (Ultra-Performance Liquid Chromatography–Electrospray Ionization Tandem Mass Spectrometry), as described in detail by Sawicki [62] and Teleszko [63]. UPLC-ESI-MS analysis was carried out using an Acquity UPLC system (Waters, Etten-Leur, The Netherlands) equipped with a BEH C18 column (2.1 × 50 mm, 1.7 μm). The column temperature was maintained at 50 °C, and the flow rate was set to 0.35 mL/min. The mobile phase consisted of 0.1% acetic acid in water (phase A) and 0.1% acetic acid in 40% acetonitrile (phase B), with a gradient elution starting at 80% A and decreasing to 50% A over 3 min.

For the analysis of anthocyanin, the mobile phases used were 2% formic acid in water (phase A) and 2% formic acid in 40% acetonitrile in water (phase B). The gradient program began with 5% B at 0 min, increased linearly to 100% B over 8 min, and was followed by a washing step and re-equilibration to initial conditions from 8 to 9.5 min. Mass spectra were acquired using a triple-quadrupole mass spectrometer (Acquity TQD, Waters) equipped with an electrospray ionization (ESI) source. Both positive and negative ion modes were applied, with a capillary voltage of 3.0 kV and a cone voltage of 50 V. Nitrogen was used as a nebulizing gas at a flow rate of 800 L/h, and desolvation was assisted by heating to 350 °C. Spectra were recorded in the mass range of 80–1100 *m*/*z*. Tandem mass spectra (MS/MS) were obtained by isolating the parent ion from direct infusion. The samples were dissolved in methanol and introduced to the ESI source using a syringe pump at a flow rate of 5 μL/min. Data acquisition and processing were performed using MassLynx 4.1 software (Milford, CT, USA). UV spectra were recorded at characteristic wavelengths: 520 nm for anthocyanins, 320 nm for phenolic acids, 360 nm for flavonols, and 280 nm for flavan-3-ols. Quantification was performed using external calibration curves prepared from phenolic standards over the concentration range of 0.05–5 mg/mL. Calibration curves showed good linearity (R^2^ ≥ 0.9998). All determinations were performed in triplicate and expressed as mg/L. The detection and identification of anthocyanins were based on specific PDA spectra, mass-to-charge ratio, and fragment ions obtained after collision-induced dissociation (CID). The quantitative analysis is based on specific MS transitions in multiple reaction monitoring (MRM) mode. The MRM transitions, cone voltage, and collision energy of each individual anthocyanin were set manually with dwell time of at least 25 ms [64].

### 3.12. Determination of Total Polyphenols and Antioxidant Properties

The total polyphenol content of the wines was determined using the method developed by Singleton [65]. Absorbance, measured 60 min after the start of the reaction, was recorded at λ = 765 nm. The procedure was repeated three times and the results were expressed as mg of gallic acid equivalent (GAE) per 1 L of wine [g GAE/L]. The ABTS^•+^ method was used to measure antioxidant potential according to Re et al. [66]. The wines, after appropriate dilution with methanol and decanting, were analyzed. The absorbance, measured at λ = 734, was recorded 6 min after the start of the reaction, using UV-1900 UV–Vis spectrophotometer (Shimadzu Corporation, Kyoto, Japan). The procedure was repeated three times and the results were expressed as mmol Trolox equivalents per liter of wine (mmol Trolox/L). The free radical neutralizing capacity of DPPH^•^ was evaluated using the method developed by Yen and Chen [67], after the wine had been previously prepared, similar to the determination of the antioxidant potential using the ABTS^•+^ method. Absorbance, measured at λ = 517 nm, was recorded 10 min after starting the reaction in the presence of 96% methanol. The procedure was repeated three times and the results were expressed in millimole of Trolox per liter of wine [mmol Trolox/L].

The FRAP test was carried out according to the spectrophotometric method presented by Benzie and Strain [68]. In total, 3 mL of FRAP solution was added to the prepared wine sample (as in the ABTS^•+^ determination). After 10 min of reaction, absorbance was recorded at 593 nm. The antioxidant activity of the wine was expressed in mmol Trolox per liter of wine [mmol Trolox/L].

### 3.13. Color Analysis

Color was determined by a CIE L*a*b* scale colorimetric method using UltraScan VIS colorimeter (HunterLab, Reston, VA, USA), in accordance with CIE 15:2004 [69] and ISO 7724/1 [70]. The colorimeter operated in the wavelength range from 360 to 780 nm, with a resolution of less than 2 nm, covering the full visible spectrum according to the CIE L*a*b* scale. The measurement was carried out in reflectance mode, using a 25 mm diameter aperture and a diffuse reflection angle of 8°. The color was measured in 10 mm thick cuvettes. Based on the data obtained, the color parameters were determined: L* (brightness, from 0-black to 100-white), a* (from −100-green to +100-red) and b* (from −100-blue to +100-yellow). Each sample was measured in triplicate.

In addition, the following indices were calculated from the average color coordinates:

Equation (1). Chroma [C*]—determining color saturation:

(1) $$C^{*}=\sqrt{a^{*2}+b^{*2}}$$

Equation (2). Hue angle [h°], which determines the dominant hue:

(2) $$h{}^{\circ}={\mathrm{TAN}}^{-1}\frac{b^{*}}{a^{*}}$$

Equation (3). Browning Index [BI]—calculated according to the formula:

(3) $$\mathrm{BI}=\left[100\times \left(x-0.31\right)\right]/0.172$$

where x is the chromaticity coordinate calculated according to Equation (4)

(4) $$x=\frac{\left(a^{*}+1.75\times L\right)}{\left(5.645\times L+a^{*}-3.012\times b^{*}\right)}$$

### 3.14. Statistical Analysis

All results are presented as mean values ± standard deviation. Statistical analysis was performed using Statistica 13.1 software (StatSoft, Tulsa, OK, USA). Differences among vino cotto samples aged for 3, 15, and 40 years were evaluated using one-way analysis of variance (ANOVA). When significant differences were found, means were compared using Fisher’s least significant difference (LSD) post hoc test at *p* ≤ 0.05. Pearson’s correlation analysis was performed to assess relationships between physicochemical parameters, phenolic compounds, free amino acids, antioxidant activity, and color parameters.

## 4. Conclusions

This study showed that vino cotto samples aged for 3, 15, and 40 years differed markedly in chemical composition, particularly in phenolic profile and antioxidant activity. These differences were related to the age of the vino cotto, although one must also take into account the potential influence of the vintage and the composition of the raw materials, production conditions, and the maturation time of wine, as well as other factors specific to individual batches. The comparison of samples suggests that higher anthocyanin contents in the older vino cotto samples may reflect retention, concentration, or formation of more stable anthocyanin-derived pigments. Color parameters also differed markedly among vino cotto samples, showing non-linear changes rather than a simple progressive darkening and browning pattern. The physicochemical characteristics and antioxidant potential of vino cotto clearly distinguished it from conventional white wine, as shown by comparison with Trebbiano d’Abruzzo. The results provide new insight into the chemical evolution of vino cotto during prolonged maturation and may support the development of production and aging strategies aimed at obtaining traditional wines with a distinctive phenolic profile and high antioxidant capacity.

## Figures and Tables

**Table 1:** Basic physicochemical parameters, sugar profile, and sulfur dioxide content of vino cotto aged for 3, 15, and 40 years and Trebbiano d’Abruzzo reference wine.

Analyte	Units	VC 3	VC 15	VC 40	*p*-Value	TDA
Density	g/cm^3^	1.04 ^a^ ± 0.00	1.06 ^c^ ± 0.00	1.05 ^b^ ± 0.00	<0.001	0.99 ± 0.00
Alcohol content	% vol.	13.35 ^a^ ± 0.00	13.60 ^b^ ± 0.08	15.88 ^c^ ± 0.10	<0.001	11.92 ± 0.11
pH		3.24 ^b^ ± 0.05	3.09 ^a^ ± 0.03	3.14 ^a^ ± 0.07	0.020	3.25 ± 0.01
Total Acidity	g tartaric acid/L	8.24 ^a^ ± 0.01	9.52 ^b^ ± 0.00	9.75 ^c^ ± 0.06	<0.001	5.77 ± 0.00
Volatile acid	g acetic acid/L	0.97 ^a^ ± 0.00	1.07 ^b^ ± 0.00	1.27 ^c^ ± 0.01	<0.001	0.33 ± 0.02
Malic acid	g/L	2.85 ^a^ ± 0.05	3.32 ^b^ ± 0.08	3.80 ^c^ ± 0.05	<0.001	1.05 ± 0.05
Fructose	g/L	9.45 ^a^ ± 0.10	11.62 ^b^ ± 0.16	14.74 ^c^ ± 0.13	<0.001	1.73 ± 0.02
Glucose	g/L	81.19 ^a^ ± 0.12	104.83 ^b^ ± 0.07	114.36 ^c^ ± 0.18	<0.001	3.40 ± 0.10
Sucrose	g/L	43.25 ^a^ ± 0.16	64.50 ^c^ ± 0.14	53.45 ^b^ ± 0.08	<0.001	3.69 ± 0.08
Total sugars	g/L	133.90 ^a^ ± 0.10	180.95 ^b^ ± 0.30	182.55 ^c^ ± 0.26	<0.001	8.82 ± 0.13
Free SO_2_	mg/L	5.82 ^a^ ± 0.20	5.47 ^a^ ± 0.18	5.41 ^a^ ± 0.17	0.068	36.13 ± 0.18
Bound SO_2_	mg/L	84.01 ^b^ ± 0.08	97.99 ^c^ ± 0.03	78.98 ^a^ ± 0.04	<0.001	97.00 ± 0.09
Total SO_2_	mg/L	89.83 ^b^ ± 0.06	103.53 ^c^ ± 0.18	84.41 ^a^ ± 0.08	<0.001	133.20 ± 0.14

Values are expressed as mean ± standard deviation. Different lowercase letters within the same row indicate significant differences among vino cotto samples aged for 3, 15, and 40 years according to one-way ANOVA followed by Fisher’s LSD test at *p* ≤ 0.05. Trebbiano d’Abruzzo was used as a reference wine and was not included in the statistical comparison unless otherwise stated. VC 3, VC 15, and VC 40: vino cotto aged for 3, 15, and 40 years, respectively. Abbreviations: SO_2_, sulfur dioxide; VC, vino cotto; TDA, Trebbiano d’Abruzzo.

**Table 2:** Free amino acid profile of vino cotto aged for 3, 15, and 40 years and Trebbiano d’Abruzzo reference wine.

Components/(mg/L)	VC 3	VC 15	VC 40	*p*-Value	TDA
Cysteine	2.90 ^b^ ± 0.13	2.67 ^b^ ± 0.07	2.16 ^a^ ± 0.19	0.029	2.90 ± 0.09
Alanine	1.56 ^b^ ± 0.04	1.37 ^a^ ± 0.03	1.55 ^b^ ± 0.03	0.014	1.46 ± 0.08
Glycine	3.49 ^a^ ± 0.06	3.61 ^a^ ± 0.06	3.25 ^a^ ± 0.30	0.265	3.51 ± 0.07
Serine	1.86 ^c^ ± 0.07	1.38 ^a^ ± 0.02	1.65 ^b^ ± 0.03	0.004	1.53 ± 0.07
Proline	35.29 ^a^ ± 0.06	37.34 ^c^ ± 0.10	36.06 ^b^ ± 0.11	<0.001	74.09 ± 0.03
Aspartic acid	1.00 ^a,b^ ± 0.01	0.96 ^a^ ± 0.04	1.09 ^b^ ± 0.04	0.047	1.18 ± 0.10
Glutamic acid	19.58 ^c^ ± 0.05	18.86 ^b^ ± 0.04	18.39 ^a^ ± 0.05	<0.001	20.05 ± 0.10
Phenylalanine	3.44 ^a^ ± 0.08	3.61 ^a^ ± 0.09	3.59 ^a^ ± 0.16	0.381	4.49 ± 0.13
Valine	0.89 ^a^ ± 0.01	0.72 ^a^ ± 0.15	0.66 ^a^ ± 0.18	0.345	1.04 ± 0.03
Threonine	3.89 ^a^ ± 0.05	3.84 ^a^ ± 0.03	3.89 ^a^ ± 0.21	0.905	3.99 ± 0.07
Leucine	0.81 ^b^ ± 0.06	0.60 ^a^ ± 0.01	0.59 ^a^ ± 0.01	0.015	0.96 ± 0.01
Lysine	10.42 ^a^ ± 0.01	11.17 ^b^ ± 0.08	11.33 ^b^ ± 0.04	<0.001	12.57 ± 0.12
Arginine	7.05 ^a^ ± 0.06	7.40 ^b^ ± 0.09	8.97 ^c^ ± 0.04	<0.001	12.92 ± 0.06
Sum of free amino acids	92.18 ^a^ ± 0.59	93.54 ^a^ ± 0.05	93.20 ^a^ ± 0.59	0.131	140.77 ± 0.40

Values are expressed as mean ± standard deviation. Different lowercase letters within the same row indicate significant differences among vino cotto samples aged for 3, 15, and 40 years according to one-way ANOVA followed by Fisher’s LSD test at *p* ≤ 0.05. Trebbiano d’Abruzzo was used as a reference wine and was not included in the statistical comparison unless otherwise stated. VC 3, VC 15, and VC 40: vino cotto aged for 3, 15, and 40 years, respectively. Abbreviations: VC, vino cotto; TDA, Trebbiano d’Abruzzo.

**Table 3:** Total polyphenol content and antioxidant activity of vino cotto aged for 3, 15, and 40 years and Trebbiano d’Abruzzo reference wine.

Parameter	Unit	VC 3	VC 15	VC 40	*p*-Value	TDA
Total polyphenols	g GAE/L	0.96 ^a^ ± 0.10	1.19 ^b^ ± 0.07	1.56 ^c^ ± 0.07	<0.001	0.61 ± 0.12
ABTS^•+^	mmol Trolox/L	0.96 ^a^ ± 0.08	9.23 ^b^ ± 0.07	13.07 ^c^ ± 0.08	<0.001	0.88 ± 0.04
DPPH^•^	mmol Trolox/L	2.14 ^a^ ± 0.09	2.44 ^b^ ± 0.02	3.68 ^c^ ± 0.04	<0.001	1.34 ± 0.10
FRAP	mmol Trolox/L	3.35 ^a^ ± 0.06	4.15 ^b^ ± 0.03	5.72 ^c^ ± 0.03	<0.001	2.00 ± 0.05

Values are expressed as mean ± standard deviation. Different lowercase letters within the same row indicate significant differences among vino cotto samples aged for 3, 15, and 40 years according to one-way ANOVA followed by Fisher’s LSD test at *p* ≤ 0.05. Trebbiano d’Abruzzo was used as a reference wine and was not included in the statistical comparison unless otherwise stated. VC 3, VC 15, and VC 40: vino cotto aged for 3, 15, and 40 years, respectively. Abbreviations: GAE, gallic acid equivalents; ABTS^•+^, 2,2′-azino-bis(3-ethylbenzothiazoline-6-sulfonic acid); DPPH^•^, 2,2-diphenyl-1-picrylhydrazyl; FRAP, ferric reducing antioxidant power; VC, vino cotto; TDA, Trebbiano d’Abruzzo.

**Table 4:** Content of quinic acid, phenolic acids, and flavonoids in vino cotto aged for 3, 15, and 40 years and Trebbiano d’Abruzzo reference wine.

	Components (mg/L)	VC 3	VC 15	VC 40	*p*-Value	TDA
*Non-phenolic organic acid*	Quinic acid	182.00 ^a^ ± 1.96	214.55 ^b^ ± 2.39	304.88 ^c^ ± 14.19	0.001	36.92 ± 1.31
*Phenolic acids*	Gallic acid	44.45 ^a^ ± 0.27	67.99 ^b^ ± 1.40	69.85 ^b^ ± 0.05	<0.001	64.26 ± 0.67
	Ethyl gallate	4.32 ^a^ ± 0.05	4.65 ^b^ ± 0.02	11.91 ^c^ ± 0.16	<0.001	4.75 ± 0.08
	Protocatechuic acid	9.24 ^a^ ± 0.16	19.14 ^b^ ± 0.74	27.45 ^c^ ± 0.21	<0.001	0.82 ± 0.00
	Caftaric acid	3.71 ^c^ ± 0.07	3.28 ^b^ ± 0.04	2.95 ^a^ ± 0.04	<0.001	10.27 ± 0.02
	Ferulic acid	0.00 ^a^ ± 0.00	0.05 ^b^ ± 0.00	0.00 ^a^ ± 0.00	<0.001	0.00 ± 0.00
	Caffeic acid	4.44 ^b^ ± 0.06	0.75 ^a^ ± 0.04	4.91 ^c^ ± 0.06	<0.001	1.50 ± 0.02
	Total phenolic acids	66.16 ^a^ ± 0.59	95.86 ^b^ ± 0.75	117.07 ^c^ ± 0.07	<0.001	81.61 ± 0.42
*Flavonoids*	Procyanidin B1	12.27 ^b^ ± 0.06	14.52 ^b^ ± 1.72	2.82 ^a^ ± 0.10	0.002	7.26 ± 0.01
	Procyanidin B2	0.04 ^a^ ± 0.02	0.42 ^b^ ± 0.03	0.69 ^c^ ± 0.02	<0.001	0.35 ± 0.06
	Procyanidin dimer	1.80 ^b^ ± 0.03	0.00 ^a^ ± 0.00	0.00 ^a^ ± 0.00	<0.001	0.36 ± 0.04
	Total flavanols	14.12 ^b^ ± 0.05	14.93 ^b^ ± 1.68	3.50 ^a^ ± 0.12	0.002	7.97 ± 0.10
	Quercetin	0.00 ^a^ ± 0.00	0.05 ^b^ ± 0.02	0.13 ^c^ ± 0.01	0.003	0.00 ± 0.00
	Quercetin 3-O-glucoside	0.01 ^a^ ± 0.01	0.01 ^a^ ± 0.00	0.02 ^a^ ± 0.01	0.713	0.00 ± 0.00
	Kaempferol 3-O-glucoside	0.16 ^a^ ± 0.01	0.16 ^a^ ± 0.01	0.18 ^a^ ± 0.02	0.596	0.15 ± 0.01
	Myricetin	0.01 ^a^ ± 0.00	0.04 ^b^ ± 0.01	0.04 ^b^ ± 0.00	0.007	0.00 ± 0.00
	Luteolin	0.36 ^b^ ± 0.04	0.00 ^a^ ± 0.00	0.00 ^a^ ± 0.00	<0.001	0.00 ± 0.00
	Total flavonols	0.55 ^b^ ± 0.05	0.26 ^a^ ± 0.00	0.37 ^a^ ± 0.03	0.009	0.15 ± 0.01
	Hesperetin	2.07 ^c^ ± 0.02	0.20 ^b^ ± 0.03	0.00 ^a^ ± 0.00	<0.001	0.94 ± 0.04

Values are expressed as mean ± standard deviation. Different lowercase letters within the same row indicate significant differences among vino cotto samples aged for 3, 15, and 40 years according to one-way ANOVA followed by Fisher’s LSD test at *p* ≤ 0.05. Trebbiano d’Abruzzo was used as a reference wine and was not included in the statistical comparison unless otherwise stated. VC 3, VC 15, and VC 40: vino cotto aged for 3, 15, and 40 years, respectively. Abbreviations: VC, vino cotto; TDA, Trebbiano d’Abruzzo.

**Table 5:** Anthocyanin profile of vino cotto aged for 3, 15, and 40 years and Trebbiano d’Abruzzo reference wine.

Components	VC 3	VC 15	VC 40	*p*-Value	TDA
Delphinidin 3-O-glucoside-5-O-glucoside	0.40 ^a^ ± 0.01	0.54 ^b^ ± 0.01	0.65 ^c^ ± 0.01	<0.001	0.00 ± 0.00
Delphinidin 3-O-glucoside	0.67 ^a^ ± 0.01	1.02 ^b^ ± 0.02	1.51 ^c^ ± 0.03	<0.001	0.00 ± 0.00
Delphinidin 3-O-(6″-O-acetyl)-glucoside	0.20 ^a^ ± 0.01	0.15 ^a^ ± 0.01	0.57 ^b^ ± 0.04	<0.001	0.28 ± 0.00
Delphinidin 3-O-(6″-caffeoyl)-glucoside	0.11 ^a^ ± 0.01	0.15 ^a^ ± 0.01	0.16 ^a^ ± 0.02	0.060	0.00 ± 0.00
Total delphinidins	1.38 ^a^ ± 0.03	1.86 ^b^ ± 0.04	2.89 ^c^ ± 0.10	<0.001	0.28 ± 0.00
Cyanidin 3-O-glucoside-5-O-glucoside	0.29 ^a^ ± 0.00	0.41 ^b^ ± 0.02	0.51 ^c^ ± 0.01	<0.001	0 ± 0.00
Cyanidin 3-O-galactoside	0.31 ^b^ ± 0.03	0.12 ^a^ ± 0.00	0.13 ^a^ ± 0.01	0.004	0.24 ± 0.00
Cyanidin 3-O-glucoside	0.00 ^a^ ± 0.00	0.38 ^b^ ± 0.00	0.93 ^c^ ± 0.01	<0.001	0.00 ± 0.00
Cyanidin 3-O-(6″-O-coumaryl)-glucoside	0.22 ^a^ ± 0.02	0.30 ^a^ ± 0.03	0.31 ^a^ ± 0.03	0.070	0.15 ± 0.00
Cyanidin 3-O-(6″-O-acetyl)-glucoside	0.41 ^a^ ± 0.01	0.41 ^a^ ± 0.00	0.44 ^b^ ± 0.00	<0.001	0.32 ± 0.00
Total cyanidins	1.22 ^a^ ± 0.00	1.62 ^b^ ± 0.04	2.33 ^c^ ± 0.05	<0.001	0.72 ± 0.01
Petunidin 3-O-glucoside-5-O-glucosid	0.68 ^a^ ± 0.02	0.73 ^b^ ± 0.02	1.03 ^c^ ± 0.03	<0.001	0.00 ± 0.00
Petunidin 3-O-glucoside	0.42 ^a^ ± 0.01	0.42 ^a^ ± 0.01	0.55 ^b^ ± 0.01	<0.001	0.25 ± 0.00
Petunidin3-O-(6″-O-acetyl)-glucoside-5-O-glucoside	0.00 ^a^ ± 0.00	0.27 ^b^ ± 0.02	0.31 ^b^ ± 0.03	0.001	0.00 ± 0.00
Petunidin 3-O-(6″-O-coumaryl)-glucoside	0.27 ^b^ ± 0.04	0.00 ^a^ ± 0.00	0.00 ^a^ ± 0.00	0.001	0.00 ± 0.00
Total petunidins	1.36 ^a^ ± 0.01	1.62 ^a^ ± 0.03	1.90 ^b^ ± 0.07	0.002	0.25 ± 0.00
Peonidin 3-O-glucoside-5-O-glucoside	0.34 ^a^ ± 0.01	0.41 ^a^ ± 0.03	0.45 ^a^ ± 0.05	0.108	0.32 ± 0.00
Peonidin 3-O-glucoside	0.38 ^a^ ± 0.03	0.39 ^a^ ± 0.02	0.50 ^b^ ± 0.04	0.045	0.31 ± 0.00
Peonidin 3-O-(6″-O-coumaryl)-glucoside	0.39 ^a^ ± 0.03	0.39 ^a^ ± 0.01	0.45 ^a^ ± 0.05	0.215	0.36 ± 0.00
Total peonidins	1.10 ^a^ ± 0.01	1.19 ^a^ ± 0.04	1.40 ^b^ ± 0.06	0.017	0.99 ± 0.00
Malvidin 3-O-glucoside-5-O-glucoside	0.61 ^a^ ± 0.03	0.66 ^ab^ ± 0.04	0.79 ^b^ ± 0.06	0.049	0.00 ± 0.00
Malvidin 3-O-glucoside	0.88 ^a^ ± 0.04	0.92 ^a^ ± 0.01	1.11 ^b^ ± 0.05	0.017	0.00 ± 0.00
Malvidin 3-O-(6″-O-acetyl)-glucoside	0.94 ^a^ ± 0.04	2.06 ^b^ ± 0.02	5.21 ^c^ ± 0.01	<0.001	0.00 ± 0.00
Total malvidins	2.43 ^a^ ± 0.06	3.64 ^b^ ± 0.07	7.11 ^c^ ± 0.10	<0.001	0.00 ± 0.00
Total anthocyanins	7.51 ^a^ ± 0.02	9.71 ^b^ ± 0.16	15.62 ^c^ ± 0.17	<0.001	2.23 ± 0.01

Values are expressed as mean ± standard deviation. Different lowercase letters within the same row indicate significant differences among vino cotto samples aged for 3, 15, and 40 years according to one-way ANOVA followed by Fisher’s LSD test at *p* ≤ 0.05. Trebbiano d’Abruzzo was used as a reference wine and was not included in the statistical comparison unless otherwise stated. VC 3, VC 15, and VC 40: vino cotto aged for 3, 15, and 40 years, respectively. Abbreviations: VC, vino cotto; TDA, Trebbiano d’Abruzzo.

**Table 6:** Color parameters of vino cotto aged for 3, 15, and 40 years and Trebbiano d’Abruzzo reference wine, determined using the CIE L*a*b* system, hue angle, chroma (C*), and browning index (BI).

Parameter	VC 3	VC 15	VC 40	*p*-Value	TDA
L*	25.74 ^c^ ± 0.08	3.69 ^a^ ± 0.21	13.20 ^b^ ± 0.15	<0.001	88.77 ± 0.04
a*	34.05 ^c^ ± 0.07	18.56 ^a^ ± 1.03	31.24 ^b^ ± 0.18	<0.001	−2.29 ± 0.01
b*	44.16 ^c^ ± 0.14	6.37 ^a^ ± 0.37	22.74 ^b^ ± 0.26	<0.001	9.31 ± 0.02
Hue angle	52.36	18.93	36.06	-	103.79
Chroma (C*)	55.76	19.62	38.64	-	9.58
Browning index	812.54	539.14	668.19	-	8.79

Values are expressed as mean ± standard deviation. Different lowercase letters within the same row indicate significant differences among vino cotto samples aged for 3, 15, and 40 years according to one-way ANOVA followed by Fisher’s LSD test at *p* ≤ 0.05. Trebbiano d’Abruzzo was used as a reference wine and was not included in the statistical comparison unless otherwise stated. VC 3, VC 15, and VC 40: vino cotto aged for 3, 15, and 40 years, respectively. Hue angle, chroma (C*), and browning index (BI) were calculated from mean L*, a*, and b* values according to Equations (1)–(4); therefore, no standard deviation or *p*-value is reported for these derived parameters. Abbreviations: L*, lightness; a*, red–green color coordinate; b*, yellow–blue color coordinate; VC, vino cotto; TDA, Trebbiano d’Abruzzo.

## Data Availability

The raw data supporting the conclusions of this article will be made available by the authors on request.
